# Hallux valgus: Advances in weightbearing CT imaging and three-dimensional assessment of first ray deformity

**DOI:** 10.1007/s00256-026-05300-5

**Published:** 2026-07-16

**Authors:** Hicham Bouredoucen

**Affiliations:** Division of Radiology, Department of Imaging and Medical Informatics, Geneva University Hospitals, University of Geneva, Rue Gabrielle-Perret-Gentil 4, 1211 Geneva 14, Geneva, Switzerland

**Keywords:** Hallux valgus, Weightbearing CT, Three-dimensional imaging, First ray deformity, Metatarsal pronation, Tarsometatarsal instability, Sesamoid complex

## Abstract

Hallux valgus (HV) is a complex triplanar deformity of the first ray that has traditionally been evaluated using weightbearing radiographs. However, these two-dimensional techniques are limited in their ability to assess the rotational and multiplanar components of the deformity. Weightbearing computed tomography (WBCT) now enables a three-dimensional assessment of the foot under physiological load, providing new insights into first ray pathology. Recent WBCT-based studies have shown that HV is part of a global malalignment of the first ray, characterized by first metatarsal pronation, medial column instability, and progressive sesamoid subluxation. These findings have refined the understanding of pathomechanical mechanisms and have influenced surgical decision-making, particularly regarding the correction of rotational deformities and instability of the tarsometatarsal joint. Emerging semi-automated tools and artificial intelligence–based methods further improve the reproducibility and efficiency of WBCT analysis. New quantitative techniques such as distance mapping and coverage mapping allow detailed assessment of articular relationships. Overall, WBCT represents a paradigm shift toward a more accurate, standardized, and comprehensive evaluation of HV.

## Introduction

Hallux valgus (HV) is a progressive triplanar deformity of the first ray, characterized by lateral deviation of the hallux, medial deviation and pronation of the first metatarsal (M1), and progressive instability of the soft tissues and osseous structures of the medial column [1]. Traditionally, HV is assessed using weightbearing radiographs, which remain the first-line imaging modality. These radiographs allow measurement of well-established angular parameters such as the HV angle (HVA), the intermetatarsal angle (IMA), and the distal metatarsal articular angle (DMAA), as well as evaluation of sesamoid position [2–4]. However, these measurements are inherently limited by their two-dimensional nature and their sensitivity to projectional variations [5, 6]. As a result, key components of the deformity—particularly axial rotation of the M1 and complex instability of the first tarsometatarsal joint (TMT) —have long been underestimated.

The development of computed tomography, and more recently weightbearing computed tomography (WBCT), has significantly improved the understanding of HV by enabling three-dimensional assessment of osseous alignment under physiological loading conditions [7–9]. WBCT allows multiplanar analysis of the first ray, revealing previously underappreciated rotational deformity, medial column instability, and alterations in metatarsosesamoid relationships. Recent studies have confirmed that HV is not solely a coronal plane deformity, but a global triplanar disorder involving coupled motion at the TMT, naviculocuneiform, and metatarsophalangeal joints [1, 10].

In parallel, advances in semi-automated segmentation and artificial intelligence (AI) have begun to improve the reproducibility and clinical applicability of WBCT-based measurements [11–13]. The aim of this narrative review is to summarize the current role of WBCT in the assessment of HV, to highlight the limitations of conventional radiography, and to present the main three-dimensional imaging parameters relevant for clinical evaluation and surgical planning.

## Epidemiology and etiopathogenic basis

HV is one of the most common forefoot deformities and represents a major issue in musculoskeletal imaging and clinical practice. A meta-analysis including 78 studies and approximately 496,000 participants reported an overall prevalence of 23% in adults aged 18 to 65 years, increasing to 35.7% after the age of 65, with a marked female predominance (30% versus 13%) [14]. From a morphological standpoint, the deformity most commonly begins in the third decade of life, although approximately one quarter of cases occur before the age of 20. Bilateral involvement is frequently observed, affecting up to 84% of patients [15]. The most common associated anatomical variations include an oval or curved metatarsophalangeal joint morphology in 71% of cases, moderate to severe metatarsus adductus in 32%, and a relatively long M1 in 71% of cases [15]. These morphological characteristics, together with female sex and a positive family history, are frequently encountered risk factors.

The etiology of HV is multifactorial and still only partially understood, involving both intrinsic and extrinsic factors [14, 16]; The role of footwear and biomechanical constraints is often suggested, although scientific evidence remains limited [17]. In contrast, a hereditary component appears more strongly supported, with family history reported in up to 90% of cases in certain series [18].

From a pathophysiological perspective, insufficiency of the medial stabilizing structures of the metatarsophalangeal joint leads to progressive medial deviation of the M1, associated with pronation of its head and secondary valgus deviation of the proximal phalanx [1]. Progression of the deformity is also driven by imbalance of the tendinous apparatus and alteration of the sesamoid complex. External mechanical constraints, particularly those related to footwear, primarily contribute to symptom expression rather than initiation of the deformity.

## Conventional weightbearing radiographic assessment of hallux valgus deformity

Conventional radiographic assessment of HV is based on weightbearing radiographs, primarily in the dorsoplantar projection, allowing global analysis of forefoot alignment and quantification of the main components of the deformity. These parameters remain essential for severity classification, despite their limitations related to their two-dimensional nature.

The main conventional radiographic parameters used in the assessment of HV deformity are summarized in Table 1.

**Table 1:** Conventional weightbearing radiographic assessment of hallux valgus deformity

Parameter	Definition and results (interpretation + key values)	Limitations/remarks
Hallux valgus angle (HVA)	Valgus deviation between the proximal phalanx and the longitudinal axis of the first metatarsal (Fig. 1a). Primary severity parameter (mild, moderate, severe). Normal < 15° [2]	Depends on axis definition and foot positioning; overall good reproducibility with digital tools [5, 6]
Interphalangeal angle	Valgus deviation between the proximal and distal phalanges of the hallux. Normal < 10° [4]. Reflects the interphalangeal contribution to overall deformity	Good interobserver reproducibility [19–21]
Intermetatarsal angle (IMA 1–2)	Angle between the axes of the first and second metatarsals (Fig. 1b). Reflects metatarsal divergence and varus of the first metatarsal. Pathologic ≥ 9° [2, 22]	Key parameter for surgical planning; sensitive to anatomical landmarks and interobserver variability [6, 21, 23]
Distal metatarsal articular angle (DMAA)	Angle between the distal articular surface of the first metatarsal and its diaphyseal axis (Fig. 1c). Normal ≤ 7° [3]	Limited reliability due to first metatarsal rotation and 2D projection; controversial in clinical practice [24–26], although correlated in severe deformities [27]
Sesamoid position	Relationship of the medial sesamoid to the axis of the first metatarsal. Graded classification systems [2, 28] or tangential analysis [29]. Reflects sesamoid complex subluxation due to pronation and medial drift of the metatarsal head [17]	Strong dependence on first metatarsal rotation and acquisition technique; intermethod discrepancies reported [28, 30, 31]. Sesamoid rotation angle (SRA) proposed as a more reproducible alternative [32]
First metatarsal (M1) rotation	Pronation of the first ray in the coronal plane, measured on axial/tangential views (or estimated on DP radiographs; WBCT via alpha angle). Increased in HV (5.7° vs 1.6° in controls) [39]	Highly sensitive to 2D radiographic technique; more accurate assessment with WBCT and 3D analysis [40]

*HV* Hallux valgus, *M1* First metatarsal, *IMA* Intermetatarsal angle, *DMAA* Distal metatarsal articular angle, *WBCT* Weightbearing computed tomography, *DP* Dorsoplantar radiograph, *SRA* Sesamoid rotation angle

### Hallux valgus angle

**Fig. 1:**
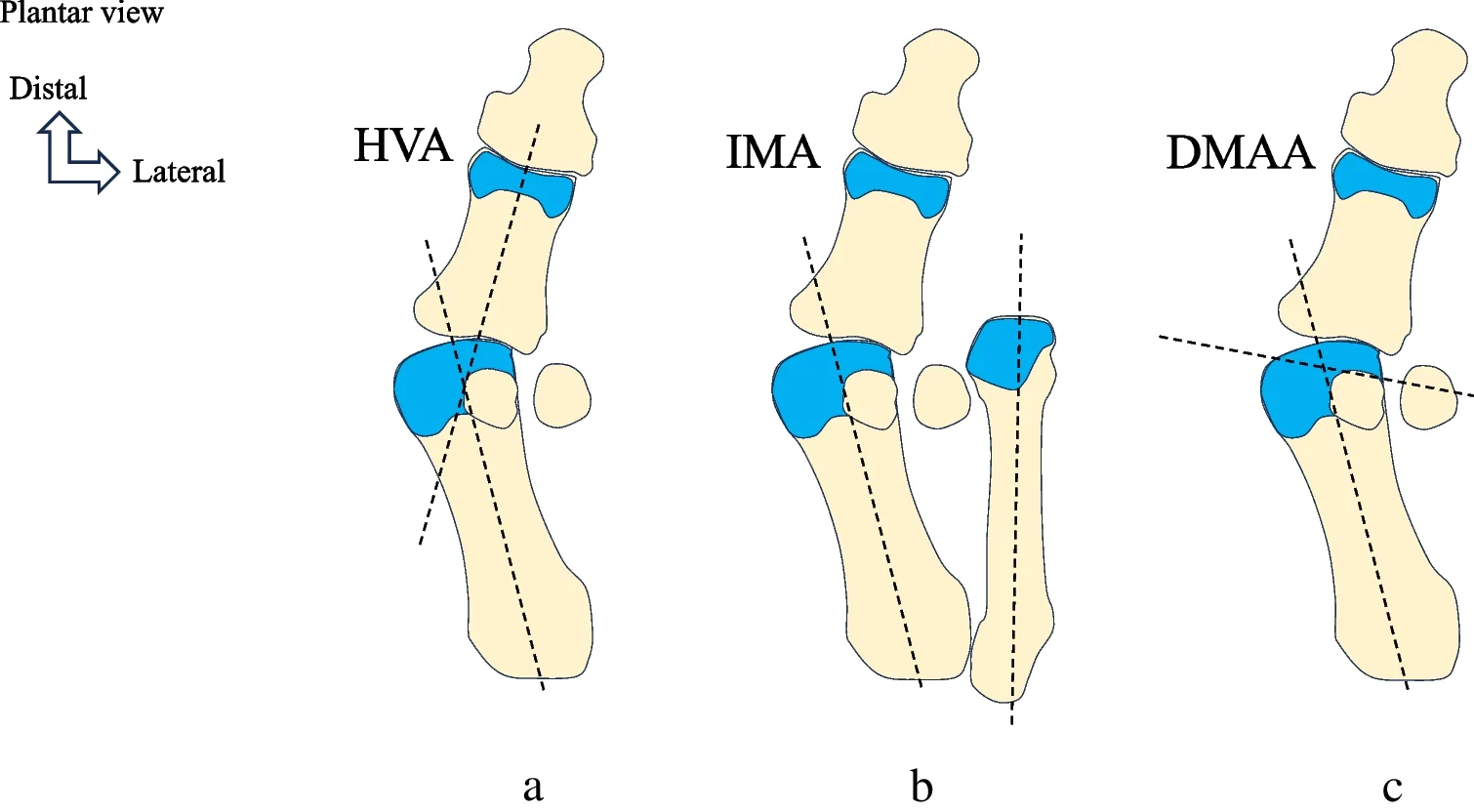
Conventional weightbearing radiographic measurements in hallux valgus. Mea**s**urement of the hallux valgus angle (HVA), defined by the axis of the first metatarsal and that of the proximal phalanx of the hallux (**a**). The intermetatarsal angle (IMA) corresponds to the angular divergence between the longitudinal axes of the first and second metatarsals (**b**). The distal metatarsal articular angle (DMAA) assesses the inclination of the distal articular surface of the first metatarsal head relative to its longitudinal axis (**c**)

The HVA corresponds to the valgus deviation of the proximal phalanx of the first ray relative to the axis of the M1 in the transverse plane, measured on weightbearing dorsoplantar radiographs (Fig. 1a) [2]. It is the main parameter used for severity stratification, generally classified into mild, moderate, and severe forms. A value below 15° is considered physiological [2]. The reproducibility of HVA measurement has been evaluated by Farber et al., who reported that interobserver agreement improved from 66% using conventional goniometric assessment to 81% with a digital workstation, while intraobserver agreement increased from 72 to 80% [5]. However, measured values remain dependent on the method used to define the first metatarsal axis. Schneider et al. demonstrated that different axis definitions may result in substantial variations in HVA measurements, with values ranging from 27.3° to 31.9° preoperatively and from 8.6° to 20.3° postoperatively depending on the technique employed [6]. These findings underscore the need for standardized measurement protocols in the radiographic assessment of HV.

### Hallux interphalangeal angle

The interphalangeal angle corresponds to the valgus deviation between the axes of the proximal and distal phalanges of the hallux, also measured on weightbearing dorsoplantar radiographs [4]. A value below 10° is usually considered normal.

Its increase reflects involvement of the interphalangeal joint in the overall deformity. HV interphalangeus has been reported in 62.1% of feet and contributes substantially to total valgus deformity of the hallux [19]. Its reproducibility has been evaluated in radiographic studies, with excellent interobserver and intraobserver reliability for hallux interphalangeal angle measurements (intraclass correlation coefficient [ICC] 0.92, 95% confidence interval [CI] 0.85–0.96, and ICC 0.94, 95% CI 0.86–0.97, respectively) [20]. In addition, HV angular measurements were reproduced within a 5° range in 86.2% of repeated assessments, supporting the reproducibility of forefoot angular measurements [21].

### First–second intermetatarsal angle

The IMA corresponds to the angulation between the longitudinal axes of the first and second metatarsals on weightbearing dorsoplantar radiographs. A value greater than or equal to 9° is considered pathological and reflects varus deviation of the M1(Fig. 1b) [2, 22]. The IMA remains a central parameter in deformity severity assessment and surgical planning. Its reproducibility is generally good, although sensitive to interobserver variability and metatarsal axis definition [6, 21, 23]. Different axis definitions can significantly affect postoperative IMA values, with reported ranges from 5.2° to 16.7° and surgical correction estimates from 0.9° to 10.0° [6]. When a standardized protocol is used, the IMA demonstrates high reproducibility, with 96.7% of repeated measurements within a 5° range [21]. The overall measurement error is approximately ± 3.6° (95% CI), with reliability coefficients ranging from 0.83 to 0.97 [23].

### Distal metatarsal articular angle

The DMAA corresponds to the angulation between the distal articular surface of the M1 and its diaphyseal axis, measured on weightbearing dorsoplantar radiographs (Fig. 1c) [3]. A value below or equal to 7° is usually considered normal. However, this parameter remains controversial as it is strongly influenced by first metatarsal rotation and radiographic projection. Its reproducibility is limited, with only 58.9% of repeated measurements falling within a ± 5° range and 22.4% differing by ≥ 8°, while interobserver agreement is lower than that reported for the first–second IMA (96.7%) and the HVA (86.2%) [21]. Measurement of the DMAA is highly dependent on first metatarsal orientation. Longitudinal rotation (*p* < 0.0001) and varus deviation (*p* < 0.02) significantly affect this angle, resulting in poor interobserver reliability (intraclass correlation coefficient = 0.14) despite good intraobserver agreement [24]. Experimental studies have demonstrated a linear increase of approximately 1.31° for every 5° increase in pronation (*p* < 0.0001), with an additional effect of metatarsal inclination (*p* < 0.0001), confirming the projection dependency of this measurement [25]. Clinical studies have also reported poor interobserver reproducibility, particularly postoperatively, with low categorical agreement (kappa coefficient = 0.07–0.19) [26]. Nevertheless, a moderate correlation between radiographic and anatomic measurements has been observed (correlation coefficient = 0.64), although only 66% of measurements fall within 5° of the true anatomic value. DMAA values increase significantly with HV severity (12.4° vs 3.2°; *p* < 0.001) [27].

### Sesamoid position and relationship with the first metatarsal head

**Fig. 2:**
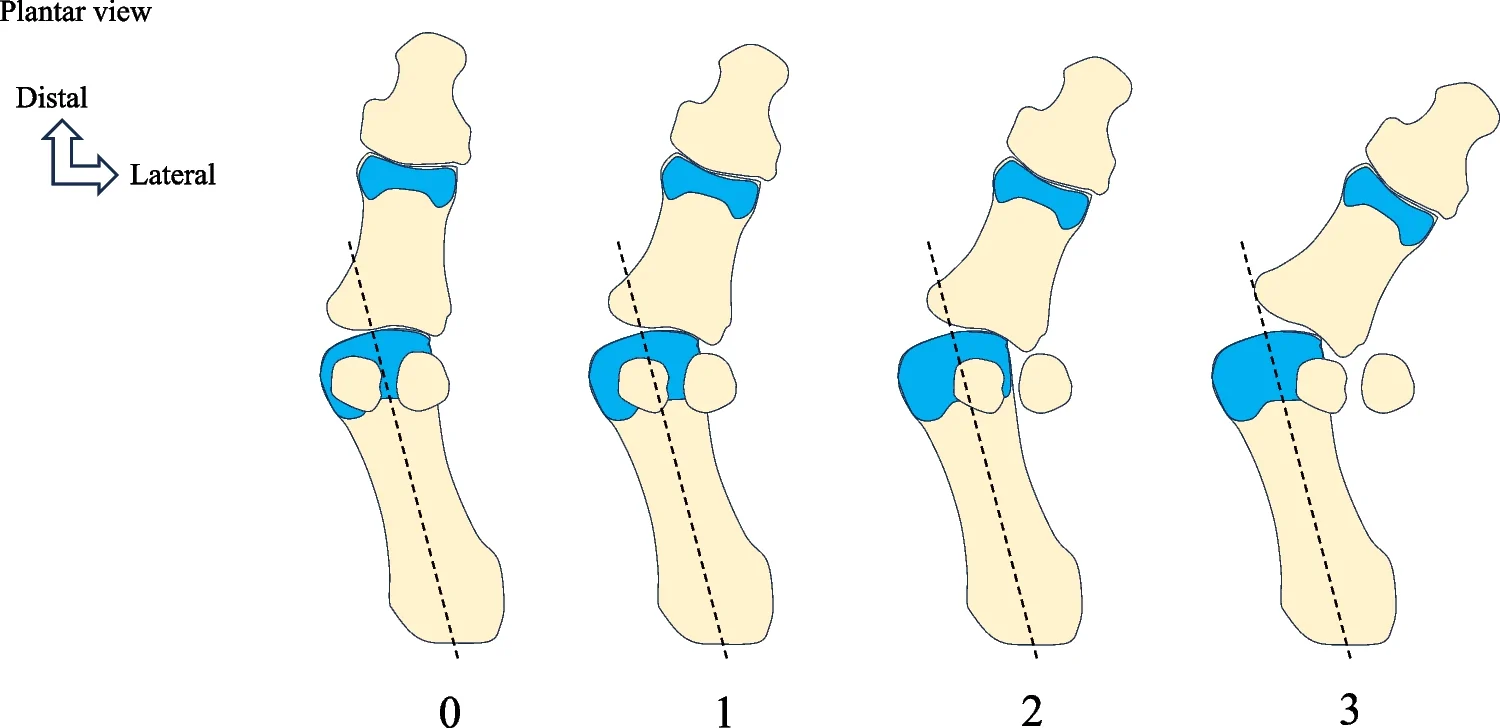
Radiographic classification of sesamoid position (AP view). Four-grade classification of sesamoid position assessed on an anteroposterior radiograph or in the transverse plane. This classification is based on the relationship between the medial sesamoid (MS) and the axis of the first metatarsal (M1). Grade 0: the MS is entirely located medial to the M1 axis. Grade 1: less than 50% overlap of the MS by the axis. Grade 2: more than 50% overlap. Grade 3: the MS is completely positioned lateral to the M1 axis

**Fig. 3:**
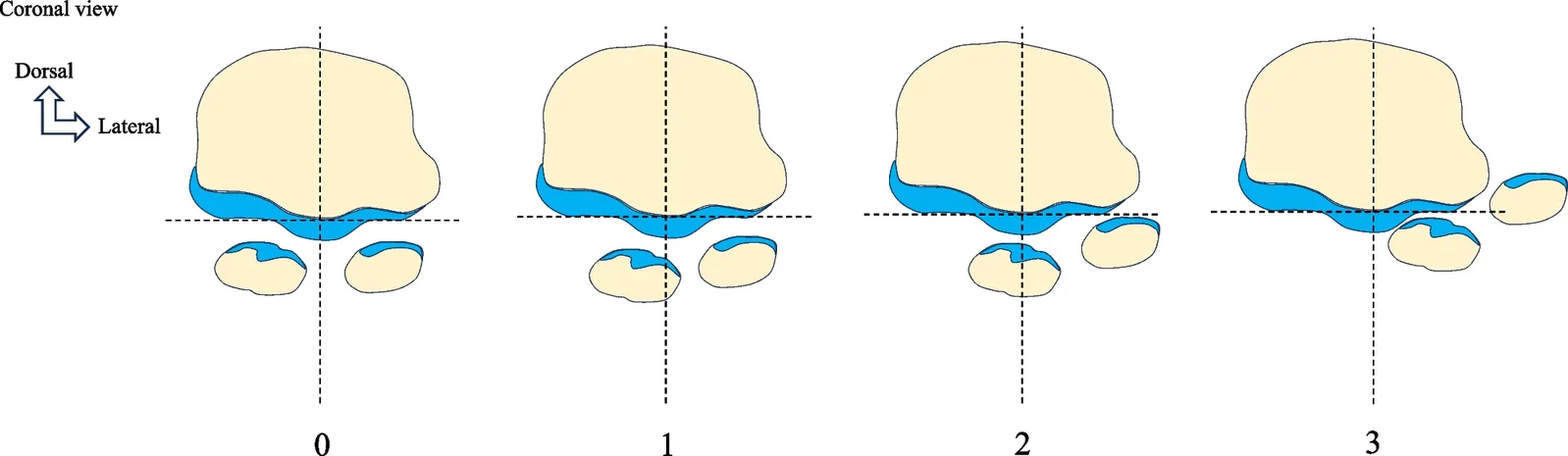
Tangential sesamoid view classification system. Four-grade classification of sesamoid position assessed on a tangential sesamoid view or in the coronal plane. This classification is based on the location of the medial sesamoid (MS) relative to the bisecting line of the intersesamoid ridge. Grade 0: the MS is entirely located medial to this line. Grade 1: less than 50% overlap of the MS with the line. Grade 2: more than 50% overlap. Grade 3: the MS is entirely located lateral to the bisecting line of the intersesamoid ridge

**Fig. 4:**
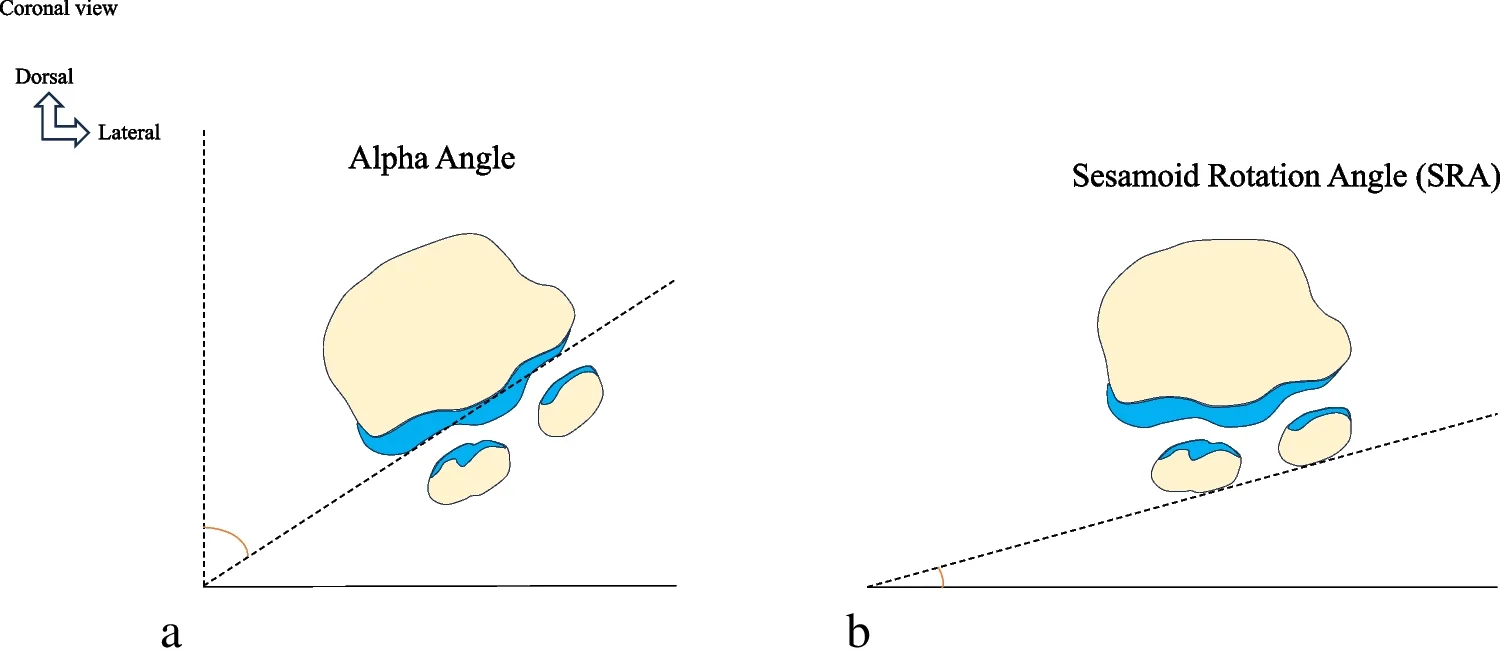
Assessment of rotational deformity on axial sesamoid view. Assessment of the rotational component on the sesamoid tangential view (coronal plane). The first metatarsal rotation angle (angle α) is defined as the angle between a line connecting the lowest points of the medial and lateral edges of the sesamoid grooves and a line perpendicular to the ground plane (**a**). The sesamoid rotation angle (SRA) is defined as the angle between the ground plane and a line tangent to the inferior aspects of the medial and lateral sesamoids (**b**)

Assessment of sesamoid position is based on analysis of their relationship to the axis of the M1 on weightbearing dorsoplantar radiographs. It allows description of progressive lateral displacement of the sesamoid complex in HV. From a pathophysiological standpoint, the head of the M1 undergoes medial migration and progressive pronation, whereas the sesamoids remain relatively fixed to the capsuloligamentous complex, explaining relative subluxation phenomena [17]. The initial Hardy classification describes seven stages of medial sesamoid displacement, later simplified into four grades ranging from complete medial position to complete lateralization (Fig. 2) [28]. In addition, the tangential view allows assessment of the relationship between the medial sesamoid and the intersesamoid ridge according to four grades of subluxation (Fig. 3) [29]. These classifications show acceptable reproducibility but remain affected by first metatarsal rotation and acquisition conditions, leading to inter-method discrepancies [28–31]. Conventional radiographic assessment of sesamoid position demonstrates moderate reproducibility, with a 95% confidence interval of approximately two grading levels between observers and limits of agreement up to 11° for angular measurements [31]. To overcome these limitations, the sesamoid rotation angle (SRA) has been proposed as a quantitative alternative for assessing sesamoid alignment (Fig. 4b) [32]. This parameter demonstrates excellent reproducibility (r = 0.98; coefficient of variation ≈2%) and a strong correlation with hallux valgus severity (r = 0.817, *p* < 0.0001), outperforming both four-grade and seven-position classifications (r = 0.769 and 0.776, respectively) [32].

### First metatarsal rotation

M1 rotation is a key component of the three-dimensional deformity in HV, corresponding to pronation of the first ray in the coronal plane [33]. It is associated with first ray hypermobility involving the TMT, naviculocuneiform, and talonavicular joints [17, 34]. Radiographic methods have also been described [35–39]. Early studies demonstrated the multiplanar nature of HV, with first metatarsal pronation correlating with intermetatarsal deformity (r = 0.69, *p* < 0.001) and medial arch collapse (r = 0.93, *p* < 0.0001) [35, 37, 38]. Tangential radiographic assessment showed good accuracy, with 5° of true rotation corresponding to 5.4° ± 1.7° radiographically and excellent interobserver reliability (r = 0.93–0.95) [39], while showing limited correlation with conventional AP angles [39]. The α angle (Fig. 4a) is a key metric for assessing first metatarsal pronation. WBCT provides a more precise three-dimensional evaluation of this parameter, which is significantly increased in HV compared with controls (21.9° ± 6.0° vs 13.8° ± 4.1°). Using a cutoff of 15.8° (upper 95% CI of controls), abnormal pronation was detected in 87.3% of HV feet [40].

## Limitations of conventional radiography and the rise of weightbearing multiplanar three-dimensional imaging

Conventional weightbearing radiographs remain the first-line imaging modality in HV due to accessibility and low cost, but their 2D nature limits evaluation of complex first-ray deformities because of projectional variability, magnification, and alignment errors, leading to incomplete assessment of rotational components and reduced reproducibility of angular measurements [9, 41, 42]. WBCT data confirm significant differences between radiographic and 3D measurements (all *p* ≤ 0.01), reflecting systematic projection-related errors despite excellent observer agreement (all *p* > 0.9) [9]. To overcome these limitations, conventional computed tomography (CT) has enabled a more precise three-dimensional analysis of osseous morphology [7, 8, 43]. However, its use in a non-weightbearing position limits the assessment of joint relationships under physiological loading conditions. Simulated weightbearing techniques have been developed, but they do not fully reproduce the biomechanical conditions of the loaded foot, particularly the action of muscular and ligamentous forces [44–47]. The major advance in recent years is represented by WBCT, which enables three-dimensional acquisition in a standing position with a lower radiation dose compared with conventional computed tomography. This technique provides detailed multiplanar analysis of osseous structures and joint relationships under conditions that are closer to true functional physiology [9, 48–51].

The development of WBCT has significantly improved the understanding of complex foot and ankle disorders, particularly multiplanar deformities such as HV and adult acquired flatfoot deformity. Beyond the limitations of conventional imaging, WBCT-based three-dimensional data have improved understanding of hindfoot–first ray biomechanics. First metatarsal pronation is closely related to medial longitudinal arch collapse and first ray laxity, resulting in load redistribution at the TMT and contributing to rotational deformity in HV [52]. A significant association has been reported between hindfoot alignment and axial rotation of the first metatarsal [53], with hindfoot valgus correlating with pronation and hindfoot varus with supination, the relationship strengthening in more severe deformities. An inverse relationship with the Meary angle further supports increased first metatarsal pronation in planovalgus deformities [53]. This coupling is consistent with the medial column biomechanical continuum, described as a rotational “zig-zag” system with intersegmental load transfer [54]. Segmental analysis shows graded medial column pronation, with higher values at the navicular (43.2°) and proximal first metatarsal (33.9°) compared with the medial cuneiform (6.1°), reflecting redistribution of rotational stress [54]. In progressive collapsing foot deformity (PCFD), hindfoot parameters are significantly associated with first ray rotation, including TMT joint involvement, with increased first metatarsal pronation in HV [55]. A proximal-to-distal increase in pronation from the navicular to the first metatarsal is also observed in planovalgus feet, reflecting transmission of rotational forces without compensatory supination [56, 57].

WBCT demonstrates high and quantifiable reproducibility, with intra- and interobserver reliability ranging from substantial to excellent. For foot deformity measurements, intraobserver ICC is ~ 0.80–0.81 (0.39–0.99), while hindfoot parameters show ICCs of 0.87–0.97 (intraobserver) and 0.51–0.88 (interobserver) [11, 12]. Morphometric validation studies report very high correlations for automated biomarkers (r up to 0.97–0.99) [58], and multi-observer analyses show good reproducibility (r ≈ 0.81–0.87) [59]. Weightbearing vs non-weightbearing WBCT shows significant differences in 18/19 variables (*p* < 0.05), including midfoot and arch parameters [11]. Method comparisons reveal dependence on reference systems, with ICCs of 0.71–0.76 and angular differences of several degrees across techniques [60]. Overall, WBCT provides a reproducible and quantitatively robust 3D assessment of foot and ankle deformities, improving diagnostic accuracy and surgical planning compared with conventional imaging [11–13, 51, 58–63].

### Weightbearing CT of the foot and ankle: radiation dose, comparative imaging, and clinical safety considerations

The selection of the most appropriate imaging modality for the diagnostic evaluation of foot and ankle disorders depends on several factors, among which safety considerations play an important role, particularly with regard to exposure to ionizing radiation (Table 2) [51, 64, 65]. Conventional CT remains a widely used technique for the assessment of osseous pathology; however, the associated radiation exposure must be considered when selecting the optimal diagnostic strategy [51].

**Table 2:** Typical effective radiation dose [51, 64, 65]

Characteristic	Dose (mSv)
Radiograph: foot (single exposure)	0.001
Radiograph: chest (p.a.)	0.02
Single transatlantic flight	0.04
WBCT: foot/ankle	0.01–0.03
Conventional CT: ankle	0.07
Tc-99 m-MDP bone scan	6.3
Average annual natural background radiation (USA)	3.0
Conventional CT: pelvis	15

*CT* Computed Tomography, *WBCT* Weight-Bearing Computed Tomography, *mSv* Millisievert (effective radiation dose unit), *p.a.* Postero-anterior projection, *Tc-99 m-MDP* Technetium-99 m methylene diphosphonate

WBCT represents a major advancement in musculoskeletal imaging, enabling image acquisition under physiological loading conditions while maintaining a relatively low radiation dose [51]. Modern WBCT systems typically deliver an effective dose ranging from 0.01 to 0.03 mSv for foot and ankle examinations, which is substantially lower than that of conventional CT and only slightly higher than, or even comparable to, standard radiographic examinations [51]. This reduction in radiation exposure, combined with the ability to assess anatomy under load-bearing conditions, contributes to the favorable overall safety profile of WBCT. Table 2 places WBCT within the spectrum of radiation exposure associated with commonly used medical imaging modalities. WBCT clearly belongs to the low-dose imaging category while simultaneously providing three-dimensional weightbearing information, a combination that largely explains its growing role in the evaluation of foot and ankle deformities.

Beyond the radiation dose of an individual examination, several studies have demonstrated that WBCT may reduce cumulative patient radiation exposure by modifying diagnostic pathways. In a large real-world study including 11,009 examinations performed in 4,987 patients, the introduction of WBCT was associated with a statistically significant reduction of approximately 10% in the mean annual effective dose per patient (4.3 µSv versus 4.8 µSv; p = 0.01) [66]. This reduction was attributed to a decreased need for multiple imaging studies, including repeated radiographic series (approximately 1.4 µSv per diagnostic episode) and conventional CT examinations (approximately 25 µSv), whereas bilateral WBCT was estimated to deliver approximately 4.2 µSv. Experimental studies have further shown that the effective dose of WBCT generally ranges from 1.9 to 14.3 µSv depending on the acquisition protocol and anatomical field of view. These values remain consistently lower than those reported for multidetector CT and may be comparable to, or even lower than, cumulative exposure from multi-view radiographic protocols when WBCT is used as a single replacement imaging modality [58]. Collectively, these findings are consistent with the principles of radiation protection and the ALARA (As Low As Reasonably Achievable) concept, supporting the favorable safety profile of WBCT in the assessment of foot and ankle disorders.

## Evolution of weightbearing CT in the definition of hallux valgus

WBCT has significantly improved the three-dimensional understanding of HV by enabling assessment under physiological load. In early WBCT studies, Collan et al. demonstrated greater deformity in HV compared with controls, with a 3D HVA of 35° (95% CI 29°–40°) versus 15° (7°–23°), and an IMA of 17° (16°–19°) versus 11° (8°–13°), respectively (*p* < 0.05) [67]. Non-weightbearing imaging showed higher values (HVA 46° vs 35° in HV; 32° vs 15° in controls), confirming load dependency [67]. Proximal phalanx pronation was markedly increased (33° ± 3° vs 4° ± 4°), while first metatarsal pronation showed a non-significant trend (8° ± 2° vs 2° ± 3°) [67]. Strong correlations were observed between 2 and 3D measurements (HVA r = 0.96; IMA r = 0.95) [67] (Table 3). WBCT kinematic studies demonstrate multiplanar instability of the TMT. During weightbearing transition, HV feet show increased dorsiflexion (2.91° ± 1.71° vs 1.18° ± 0.47°, p = 0.001), supination (2.17° ± 2.28° vs 0.98° ± 0.81°, p = 0.045), and internal rotation (2.65° ± 2.22° vs 0.96° ± 0.57°, p = 0.006), with joint widening (p = 0.030) [68]. The α angle is significantly increased in HV (21.9° ± 6.0° vs 13.8° ± 4.1°), exceeding 15.8° in 87.3% of cases [40]. Correlations with radiographic parameters remain weak (HVA ρ = 0.076; IMA ρ = 0.144; sesamoid ρ = 0.019), with radiograph–CT disagreement in 39.7% and pseudo-subluxation in 25.9% [40], supporting WBCT-based classification systems (Fig. 3).

**Table 3:** Evolution of WBCT in the three-dimensional understanding of hallux valgus

WBCT parameters analyzed	Main findings	Key contribution
HVA, IMA (2D/3D), first ray rotation	HVA and IMA increased in hallux valgus (HV); higher HVA in non-weightbearing conditions; increased proximal phalanx pronation; strong correlation between 2 and 3D measurements [67]	Initial validation of WBCT for comprehensive 3D assessment
TMT kinematics (3D)	Increased dorsiflexion, supination, and internal rotation of the TMT joint; multiplanar hypermobility [68]	Evidence of TMT hypermobility in HV
Alpha angle, sesamoids	Increased alpha angle; weak correlation between HVA/IMA and WBCT findings; sesamoid incongruence [40]	Limitations of 2D radiographs and added value of WBCT
Multiaxial first ray kinematics	Global hypermobility of TMT, naviculocuneiform (NC), talonavicular (TN), and MTP1 joints; strong correlation with HVA/IMA [10]	HV as a multiplanar global deformity
Intercuneiform interactions	Increased dorsiflexion and inversion of the intermediate cuneiform [69]	Extension of medial column hypermobility
M1 and proximal phalanx pronation	Increased pronation; weak radiographic correlation [70]	Dissociation between rotation and 2D radiographic measures
Sesamoid kinematics	Increased SRA; sesamoid displacement; association with increased HVA/IMA [1]	WBCT-based classification (Stanmore system)
Alpha angle and M1 morphology	Increased alpha angle; weak correlation with radiographic morphology [71]	3D rotational deformity independent of 2D morphology
M1 and sesamoid rotation	Increased M1 pronation and sesamoid rotation; global torsional deformity [72, 73]	Confirmation of a global 3D deformity pattern
Hindfoot–forefoot alignment and M1 rotation	Biomechanical coupling between hindfoot and forefoot [52, 53, 74]	Medial column biomechanical continuum
M1 rotation and TMT	Global pronation; independent HV–M1–TMT association [55]	Confirmed link between PCFD and HV
Rotational patterns	Similar M1 pronation patterns; differences in TMT/navicular involvement [57]	Phenotypic differentiation
Medial column behavior	“Zig-zag” pattern in HV vs homogeneous pronation in PCFD [54, 56]	Distinct biomechanical models
M1 pronation and clinical scores	Pronation correction associated with improved PROMIS scores and reduced recurrence [76]	Clinical relevance of rotational deformity

*WBCT* Weightbearing computed tomography, *HV* Hallux valgus, *HVA* Hallux valgus angle, *IMA* Intermetatarsal angle, *TMT* tarsometatarsal joint, *NC* Naviculocuneiform joint, *TN* talonavicular joint, *MTP1* First metatarsophalangeal joint, *M1* First metatarsal, *SRA* Sesamoid rotation angle, *PROMIS* Patient-Reported Outcomes Measurement Information System, *PCFD* Progressive collapsing foot deformity

Medial column instability is consistently demonstrated. Increased talonavicular dorsiflexion (3.9° ± 2.2° vs 2.1° ± 1.6°, p = 0.043) and cuneonavicular eversion (1.5° ± 1.0° vs 0.2° ± 1.5°, p ≈ 0.039) indicate proximal hypermobility. At the first TMT joint, dorsiflexion, inversion, and adduction are increased (3.6° ± 2.3° vs 2.0° ± 1.3°; 4.9° ± 3.6° vs 2.6° ± 1.4°; 3.2° ± 2.3° vs 1.1° ± 0.7°, all *p* < 0.05) [10]. Distal propagation is reflected by increased eversion and abduction at the first MTP joint. Radiographic correlations can reach r = 0.98 but fail to capture 3D kinematics [10]. Selective intercuneiform involvement is observed at the 1–2 joint, with increased dorsiflexion (0.8° ± 0.6° vs 0.1° ± 0.3°, p = 0.0067) and inversion (1.0° vs 0.2°, p = 0.0019) [69]. First ray pronation is consistently increased in HV, with first metatarsal rotation (27.3° ± 10.6° vs 19.1° ± 7.1°, p ≈ 0.048) and proximal phalanx pronation (12.9° ± 17.3° vs − 1.6° ± 3.6°, p ≈ 0.013) [70]. Sesamoid assessment shows excellent reproducibility (ICC 0.960–1.000) [1], with rotation reaching 15–20° and progressive joint narrowing. A WBCT classification integrating sesamoid position shows significant differences (*p* < 0.0001) (Fig. 3) [1]. Radiographic morphology is limited, as the “round head sign” shows a weak correlation with the α angle (R^2^, coefficient of determination, ≈ 0.15), whereas sesamoid position is more predictive (R^2^ ≈ 0.37). WBCT confirms increased first ray rotation (11.5° vs 4.2°) and increased sesamoid rotation (26.7° vs 4.6°) [71]. Intrinsic first metatarsal torsion is significantly increased (~ 15° vs 3–4°), with global torsion reaching ~ 30° in severe HV (*p* < 0.001) [72]. Randich et al. reported higher sesamoid rotation (23.5° vs 6.6°), metatarsal head rotation (17.8° vs 9.8°), and torsion (22° vs 13.5°), without TMT differences, suggesting a distal origin [73].

**Fig. 5:**
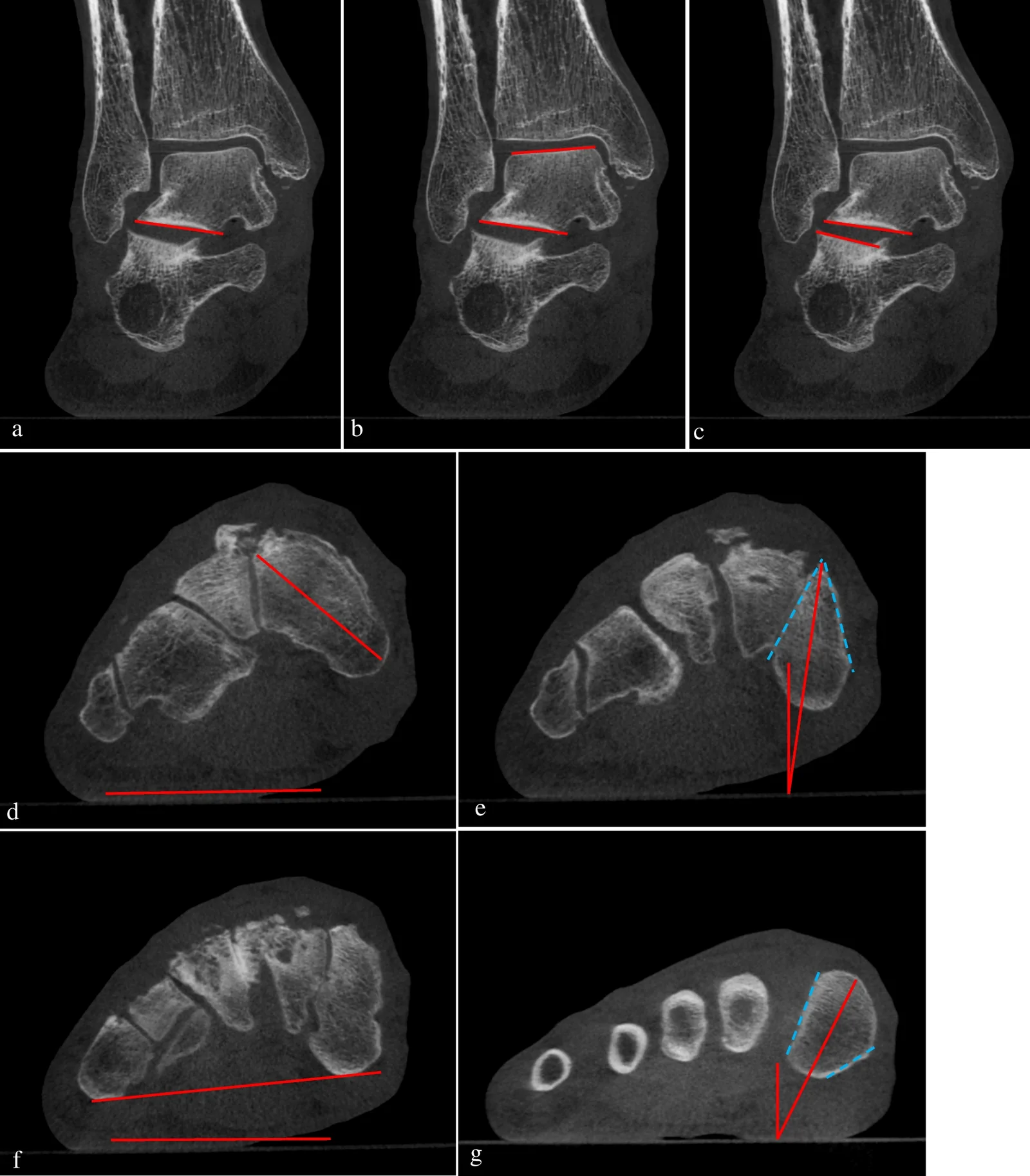
Three-dimensional manual rotational measurements of the medial column in WBCT. Medial column rotational parameters manually measured on WBCT. Blue dashed lines represent tangents to osseous surfaces used for measurements. Red lines correspond to the rotational parameters assessed. (**a**) Subtalar horizontal angle. (**b**) Infratalar–supratalar angle. (**c**) Infratalar–supracalcaneal angle. (**d**) Navicular rotation. (**e**) Medial cuneiform rotation. (**f**) Forefoot arch angle. (**g**) First metatarsal base rotation

A coupled hindfoot–midfoot–forefoot system is also evident. First ray rotation ranges from ~ 5° in normal feet to 10–20° in deformity [58], with correlation to hindfoot alignment (r ≈ 0.62–0.64) [53]. Flatfoot correction may increase HVA (~ 4°), with progression ≥ 5° in 50% and talonavicular coverage < 21.6° predictive [53, 74]. In progressive collapsing foot deformity, WBCT shows coupled rotation (R^2^ ≈ 0.61) (Fig. 5) [55]. Distinct medial column phenotypes include a “zig-zag” pattern (navicular 43.2°, M1 33.9°, cuneiform 6.1°) [54]. HV combines M1/TMT pronation with naviculocuneiform supination (− 9.7°), while collapsing foot deformity shows more homogeneous pronation [56, 57].

Clinically, DMAA decreases from 25.9° to 11.9° (~ − 14°) after correction, indicating radiographic overestimation [75], with improved PROMIS (+ 7) and reduced recurrence (11.5% vs 46.2%) [76]. Crista degeneration reduces pronation (~ 16° vs ~ 29°) [77]. Overall, WBCT demonstrates that HV is a complex three-dimensional first ray deformity combining metatarsal pronation, sesamoid malalignment, medial column instability, and hindfoot coupling, which are incompletely captured by radiographs, supporting its role in pathophysiological understanding and surgical planning.

## Weightbearing CT: main parameters in hallux valgus

WBCT enables a three-dimensional assessment of HV that goes beyond the limitations of conventional radiographic measurements. It provides a more detailed characterization of TMT instability and rotational deformities of the M1, integrating combined analysis of the sagittal, axial, and coronal planes. Initial two-dimensional assessments of the tarsometatarsal and midfoot complex have been extended to biplanar radiographs and WBCT, enabling multiplanar evaluation of first ray instability in HV [78–80]. Across modalities, consistent abnormalities include midfoot malalignment with increased talonavicular coverage and lateral talo–first metatarsal angles, reflecting a planovalgus pattern [78]. Sagittal instability is evidenced by increased dorsal translation and dorsiflexion at the first TMT or MC1 joint, from ~ 2 mm and ~ 2° on radiographs to ~ 1.15° TMT angle increase on WBCT, versus near-zero values in controls [78, 79]. WBCT demonstrates axial and rotational deformity, with increased C1–M1 angle (~ 29° vs 22°) and plantar gapping (~ 1.1 vs 0.37 mm), confirming instability [79]. Distal first metatarsal pronation is increased (~ 17.9° vs 7.1°), whereas proximal rotation shows no difference, indicating distal predominance [79]. CT analyses confirm increased pronation, with α angles (~ 21.9° vs 13.8°) and metatarsal pronation angles (~ 6–11° vs 2°) [80], with high inter- and intraobserver ICCs (0.83–0.99; 0.93–0.96) [80]. Overall, WBCT provides robust quantitative assessment of first ray deformity [78–80].

The key WBCT parameters used to assess HV across the sagittal, axial, and coronal planes are summarized in Table 4.

**Table 4:** WBCT assessment of hallux valgus: key parameters by anatomical plane

Plane	WBCT parameters (interpretation + findings)	Clinical implications
Sagittal	First TMT angle ↑ (HV 1.9° vs controls 0.9°), reflecting articular incongruence between the medial cuneiform (C1) and the first metatarsal (M1); plantar distance ↑ (HV 1.14 mm vs controls 0.37 mm), indicating loss of plantar support of the first ray; dorsal translation of M1 present in ~ 63% of HV cases, suggesting dorsal subluxation; sagittal TMT version not significant [78, 79, 81]	Sagittal TMT instability with loss of plantar coaptation and a tendency toward dorsal translation of the first metatarsal
Axial	HVA and IMA ↑ in HV vs controls, reflecting hallux valgus deformity and metatarsal divergence; C1–M1 angle ↑ (HV 29.5° vs controls 22.4°), indicating malalignment of the first ray; C2–M2 angle used as a marker of metatarsus adductus; axial TMT version not significant [79, 82, 83]	Global forefoot malalignment with axial instability of the first ray
Coronal	Alpha angle ↑ (HV 21.9° vs controls 13.8°; ~ 87% > 16°), indicating pronation of the metatarsal head; metatarsal pronation angle (MPA) ↑ (HV ~ 6° vs controls ~ 2°), reflecting first metatarsal rotation; sesamoid rotation ↑ (HV ~ 29.2° vs controls ~ 9.7°), indicating sesamoid complex subluxation; metatarso-sesamoid rotation ↑ (preoperative HV ~25.3°, reduced to ~2.6° postoperatively), indicating restoration of joint congruency after surgery [1, 40, 67, 84, 85]	Major rotational deformity of the first ray involving the metatarso-sesamoid complex

*WBCT* Weightbearing computed tomography, *HV* Hallux valgus, *TMT* Tarsometatarsal joint, *C1* Medial cuneiform, *M1* first metatarsal, *C2* Intermediate cuneiform, *M2* Second metatarsal, *HVA* Hallux valgus angle, *IMA* Intermetatarsal angle, *MPA* Metatarsal pronation angle

### Sagittal plane analysis

**Fig. 6:**
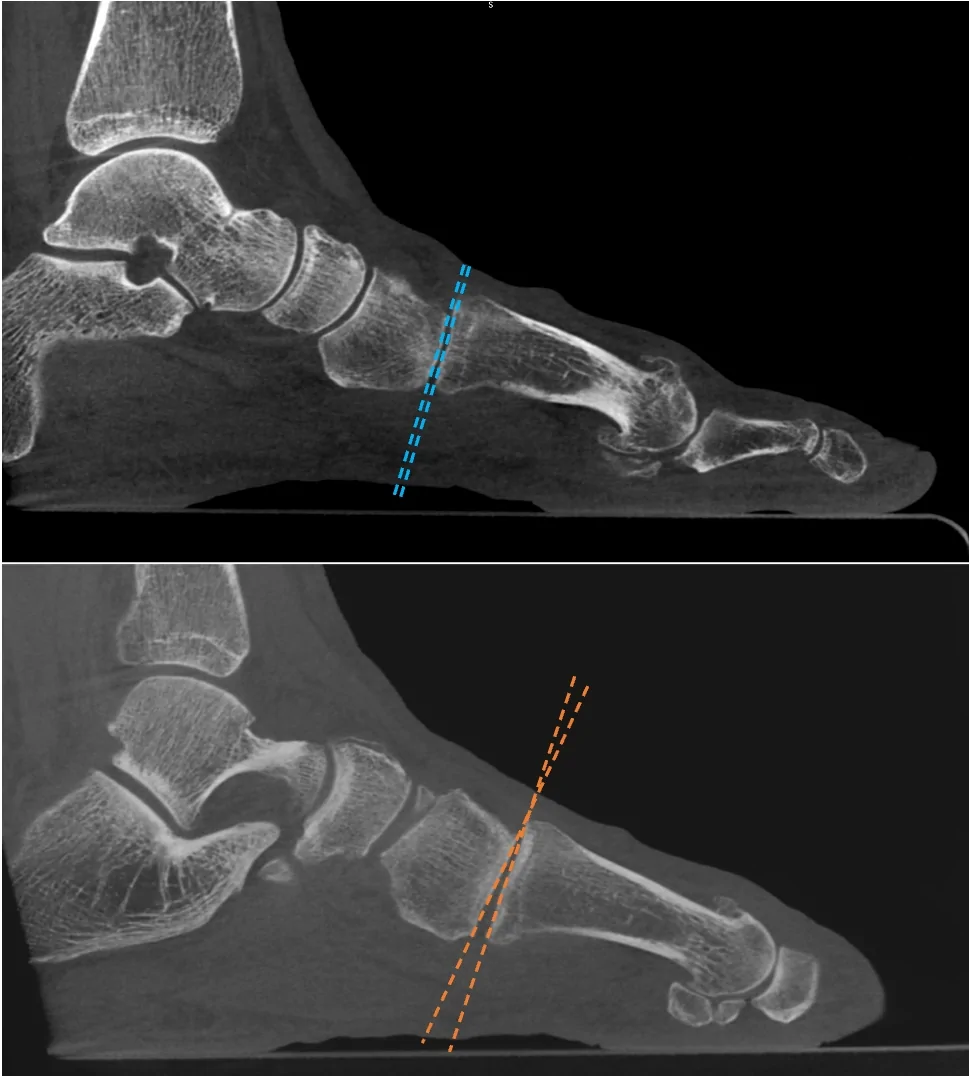
Tarsometatarsal joint angle measurement (medial cuneiform–first metatarsal angle). The tarsometatarsal (TMT) joint angle is defined as the angle formed by two lines drawn along the articular surfaces of the medial cuneiform (C1) and the first metatarsal (M1), respectively, at the mid–mediolateral level of the TMT joint

**Fig. 7:**
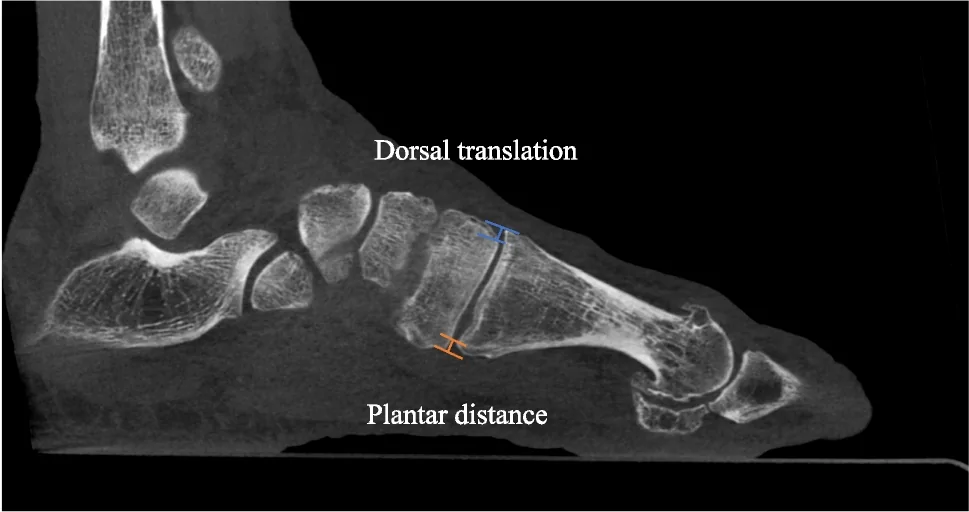
Dorsal translation and plantar joint distance at the TMT joint. Dorsal translation of the first metatarsal (M1): at the TMT joint, defined as a step-off or discontinuity of curvature along the plantar or dorsal contours of the C1 and M1. Plantar joint distance of the TMT joint: defined as the vertical distance between the lowest point of the distal articular surface of the medial cuneiform (C1) and the lowest point of the proximal articular surface of the first metatarsal (M1), measured at the mid–mediolateral level of the TMT joint

**Fig. 8:**
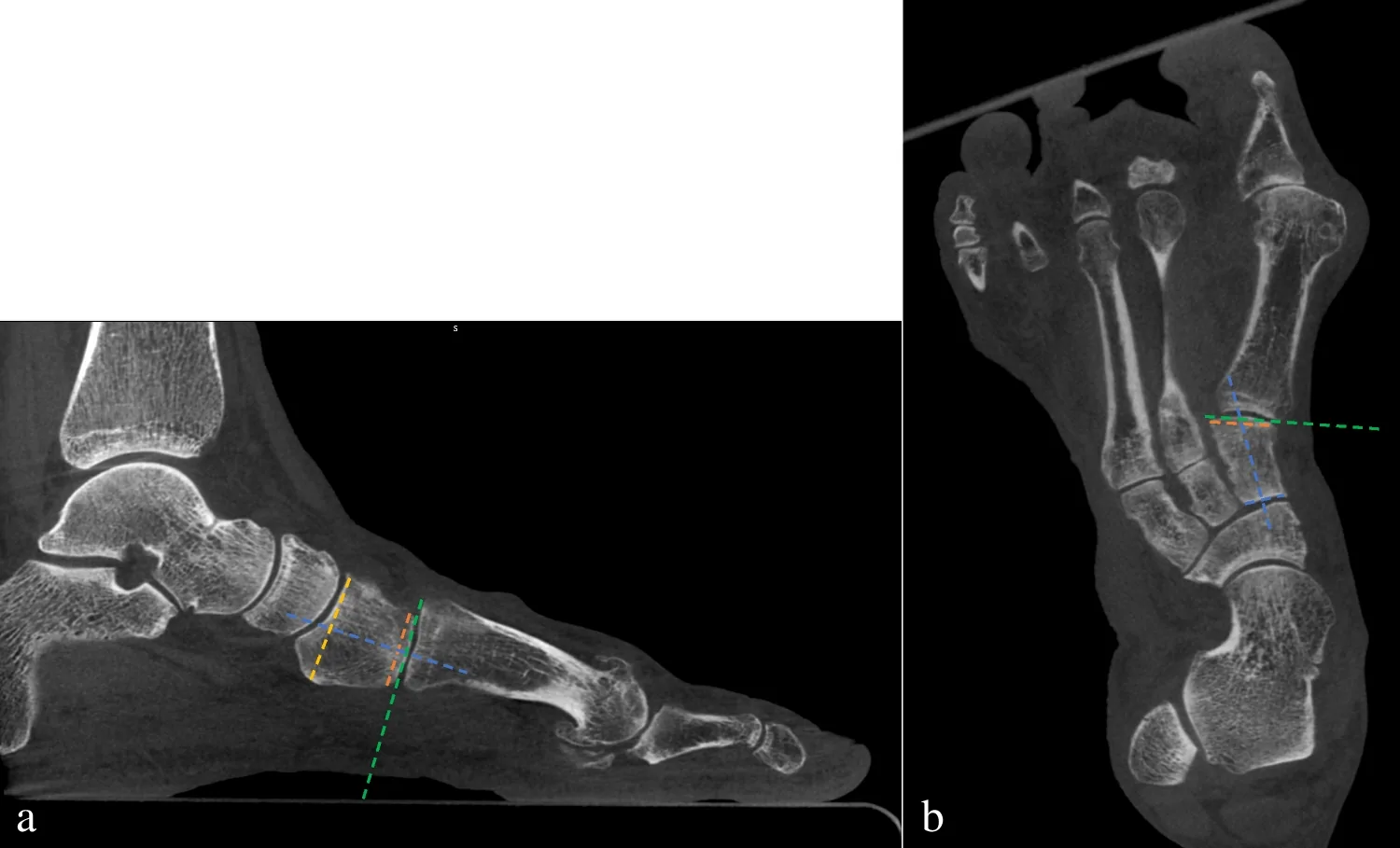
Sagittal and axial alignment parameters of the first tarsometatarsal joint. (**a**) Sagittal TMT version: angle between the longitudinal axis and the distal articular surface of the medial cuneiform (C1). (**b**) Axial TMT version: defined as the angle measured in the axial plane between the longitudinal axis and the distal articular surface of the medial cuneiform (C1)

Sagittal instability of the TMT is quantified on WBCT using the sagittal TMT angle between the medial cuneiform (C1) and the first metatarsal (M1). In the systematic review by Talaski et al. (2024), this parameter was increased in HV, with a frequency-weighted mean of 1.9 ± 1.4° versus 0.9 ± 0.7° in controls, confirming sagittal midfoot instability (Fig. 6) [81]. Sagittal plane assessment of the first TMT joint shows consistent first ray instability in HV. Early radiographic studies reported dorsal translation of 2 mm versus 0 mm in controls and dorsiflexion of 2° at the TMT joint [78]. WBCT shows increased plantar C1–M1 distance (1.14 vs 0.37 mm) (Fig. 7) [79]. Dorsal M1 translation is more frequent (63% vs 23%) [79], supporting dynamic instability. The first TMT angle is increased (1.15° vs 0.23°) [79], while overall sagittal alignment does not differ significantly [78, 79], indicating that instability is better captured by translation and gapping parameters than by global orientation. Conversely, sagittal version of the TMT joint shows no significant difference between patients and controls (Fig. 8a) [78, 79].

### Axial plane analysis

**Fig. 9:**
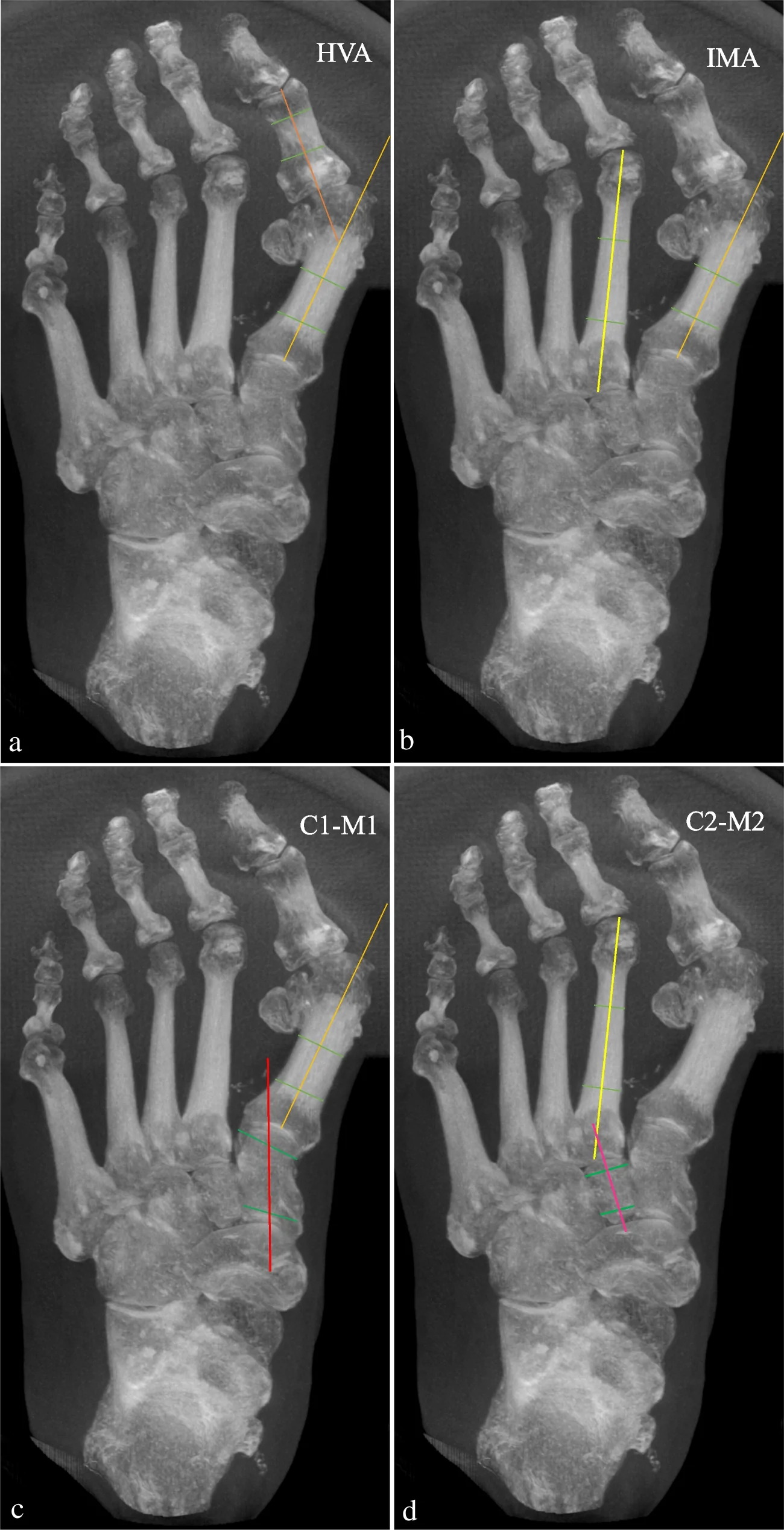
Axial WBCT assessment of forefoot alignment in hallux valgus**.** Axial view showing HVA (**a**), IMA (**b**), C1–M1 angle (**c**), and C2–M2 angle (**d**)

In the axial plane, weightbearing imaging (radiography and WBCT) demonstrates complex forefoot malalignment in HV, with HVA and IMA as key severity indicators (Fig. 9). Radiography showed increased talonavicular coverage, indicating midfoot abduction [78]. WBCT confirms multiplanar deformity with increased HVA and IMA (33.5° vs 7.5°; 16.98° vs 9.46°, *p* < 0.001) [79, 82]. The C1–M1 angle is increased (29.46° vs 22.44°, *p* < 0.001) [79, 82], while the C2–M2 angle reflects metatarsus adductus [83]. TMT axial version (Fig. 8b) and proximal rotation show no differences [78, 79]. Distal metatarsal pronation is increased (17.88° vs 7.06°), supported by higher α angles and more cases above 16° [79, 82]. Calcaneal pitch is unchanged, indicating forefoot-confined deformity [82].

### Coronal plane analysis

The coronal plane is the main site for rotational assessment of the first ray.

**Fig. 10:**
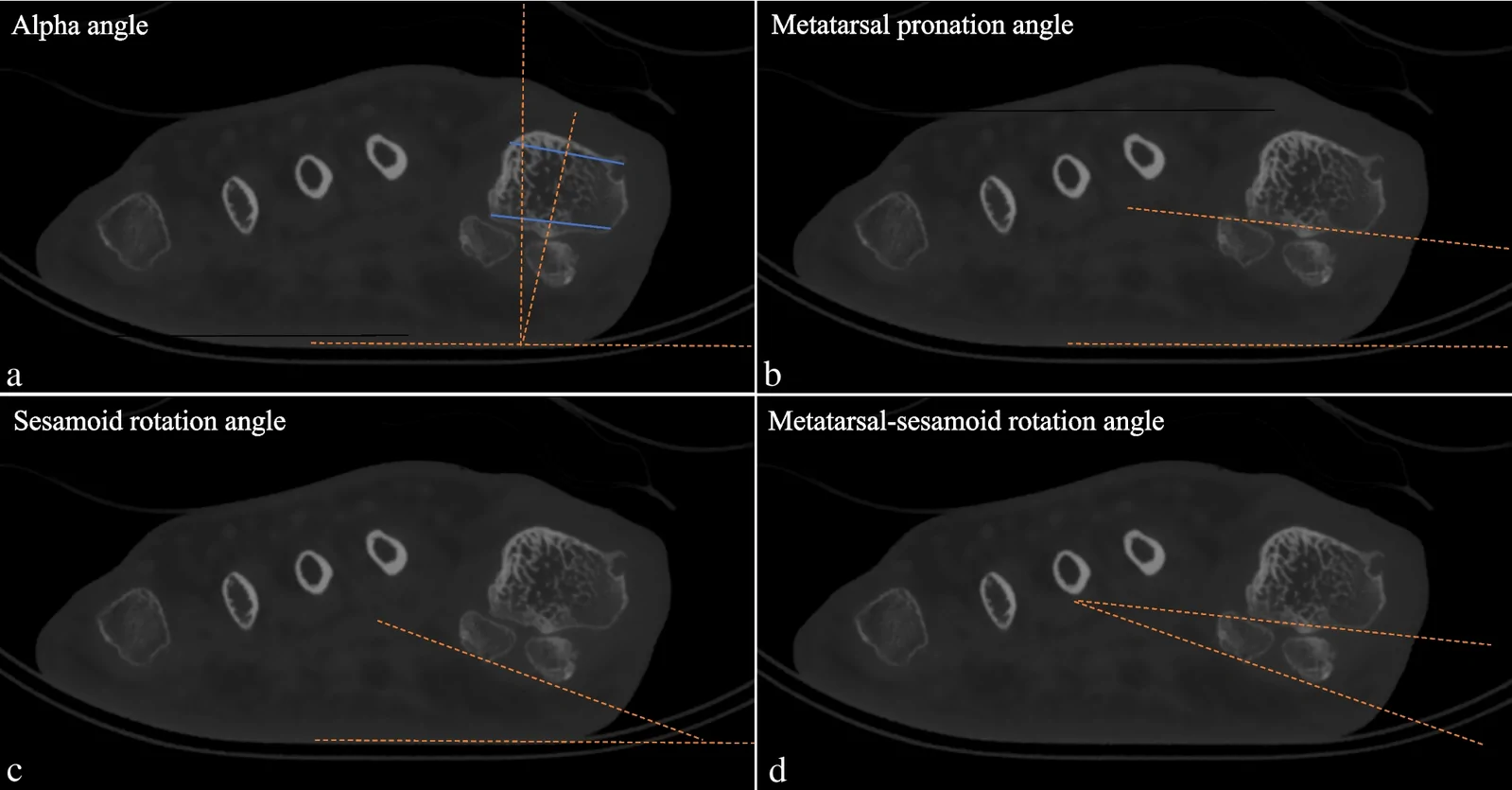
Coronal WBCT rotational assessment of the first ray. Coronal view showing alpha angle (**a**), metatarsal pronation angle (**b**), sesamoid rotation angle (**c**), and metatarsal–sesamoid rotation angle (**d**)

The alpha angle (Fig. 10a) and the metatarsal pronation angle (MPA) (Fig. 10b) quantify first-ray pronation in HV but at different anatomical levels [40, 67, 79, 84]. The alpha angle is measured at the metatarsal head using lines drawn along the medial and lateral sulcus margins and the medial and lateral corners of the head, with projection relative to a vertical axis to define rotational deviation [40, 84]. The MPA is derived from the sesamoid articular facets, using a line along the inferomedial and lateral margins of the facets and a second line along the weight-bearing surface; the angle between these lines reflects overall first-ray pronation under load conditions [67, 84]. From a biomechanical perspective, HV combines distal metatarsal rotation and proximal tarsometatarsal instability [79, 84]. The alpha angle is more sensitive to distal morphological changes but is highly dependent on precise landmark definition, which may limit reproducibility [40, 84], whereas the MPA relies on broader osseous landmarks and demonstrates higher reliability in WBCT-based assessments [67, 84]. Lee et al. [79] showed that rotational deformity varies according to the anatomical level assessed, indicating that local measurements capture only part of the global deformity. Scheele et al. [84] further demonstrated that surgical correction affects metatarsal rotation and sesamoid alignment differently, supporting the need for a global assessment of pronation.

Accordingly, the alpha angle primarily reflects distal metatarsal head torsion, whereas the MPA reflects overall first-ray pronation under load conditions. These parameters therefore assess complementary but distinct aspects of the deformity and should not be considered interchangeable, with the MPA appearing more robust for functional alignment assessment [40, 67, 79, 84]. The alpha angle is increased in HV, from 13.8° ± 4.1° in controls to 21.9° ± 6.0° in affected feet [40]. Using 15.8° as a threshold, pathological pronation was present in 87.3% of HV feet [40]. Proximally, phalanx pronation is increased (33° vs 4°, *p* < 0.05), whereas first metatarsal pronation is not significantly different (8° vs 2°, p > 0.05) [67]. Distal metatarsal rotation is increased (17.9° vs 7.1°, *p* < 0.001), while proximal metatarsal and medial cuneiform rotation show no significant differences [79].

WBCT confirms coronal plane deformity beyond conventional radiographs. The MPA decreases after Lapidus procedure (11.4° to 5.6°), with pathological pronation (> 10°) decreasing from 56.7% to 36.7% [84]. These changes are associated with + 3.1 mm elevation of the second metatarsal head and − 3.8 mm lateral displacement of the sesamoids [84]. The MPA shows no correlation with HVA or IMA [84]. WBCT demonstrates strong reliability for HVA (r = 0.94–0.96) and IMA (r = 0.81–0.95) [67]. Sesamoid rotation (Fig. 10c) is increased, with excellent reproducibility (ICC = 0.991) and strong discriminative ability (*p* < 0.0001) [1]. It varies according to position: 10.6° ± 5.1° in supination versus 22.2° ± 7.7° in pronation, with intermediate radiographic values (12.3° ± 9.5°) [85]. Metatarsal–sesamoid rotation (Fig. 10d) is approximately 25°, decreasing postoperatively in parallel with a reduction in pronation prevalence (56.7% to 36.7%) [84]. These changes include − 3.8 mm sesamoid and + 3.1 mm metatarsal displacement [84], without correlation with HVA or IMA. Overall, WBCT provides a reproducible and quantitative assessment of rotational deformity that is substantially underestimated by conventional radiography.

### Reported values and suggested thresholds for first metatarsal pronation (α angle/MPA) on 3D WBCT

Studies using weight-bearing three-dimensional imaging (CT or CBCT) report different measurements of first metatarsal pronation, with no consensus regarding a universal threshold for normality or pathology; therefore, values must be interpreted according to the specific methodologies used in each study. Kim et al. (2015) [40], using the α angle on semi-weight-bearing CT, reported a mean value of 13.8° ± 4.1° in controls, with an upper 95% confidence interval limit of 15.8° used as a discriminative threshold, compared with 21.9° ± 6° in patients with HV. Collan et al. (2013) [67] assessed the MPA on WBCT and reported values close to 0–2° in asymptomatic subjects and approximately 8° in HV. Lee et al. (2022) [79] reported a WBCT MPA of 7.06° in controls and 17.88° in HV, confirming a significant increase in pronation. Scheele et al. (2020) [84] described a CBCT MPA of 11.4° preoperatively and 5.6° after Lapidus arthrodesis, with a pathological threshold defined as > 10°. Thus, despite the absence of a universal threshold, MPA values converge toward physiological values close to 0–2° and a pathological threshold > 10°, whereas the discriminative threshold for the α angle is 15.8°. These data are summarized in Table 5.

**Table 5:** Reported values and pathological thresholds for first metatarsal pronation (α angle/metatarsal pronation angle [MPA]) on 3D WBCT

Parameter	Control/asymptomatic	Hallux valgus + pathological threshold
α angle [40]	13.8° ± 4.1° (upper 95% CI: 15.8°)	21.9° ± 6°; ≥ 15.8° = pathological threshold (95% CI of controls)
MPA [67]	~ 0–2°	~ 8°
MPA [79]	7.06°	17.88°
MPA [84]	5.6° postoperative	11.4° preoperative; > 10° = pathological pronation

*α angle* First metatarsal pronation angle, *MPA* Metatarsal pronation angle, *CI* confidence interval

## Therapeutic implications and surgical perspectives

Surgical management of HV now incorporates the three-dimensional nature of the deformity, particularly the rotational component of the M1, as well as the biomechanical insufficiency of the first ray associated with instability of the TMT and medial arch collapse [86]. In this context, the biomechanical interactions between the hindfoot and forefoot raise important questions regarding the appropriateness of isolated forefoot correction in the presence of proximal malalignment. The therapeutic implications of this relationship remain incompletely understood [53]. Compensatory mechanisms in planovalgus and cavovarus deformities are well established [53], and rotational abnormalities of the first metatarsal should be integrated into complex reconstructive strategies. However, it remains unclear whether isolated correction of hindfoot malalignment is sufficient to restore forefoot morphology, or whether each component requires targeted management [53]. The medial column has been described as a continuous biomechanical unit with a chain-like rotational organization and consistent intersegmental relationships [54]. In progressive collapsing foot deformity (PCFD), increased pronation is observed throughout the entire medial column, from the navicular to the first ray, without compensatory supination, reflecting a continuous pattern of rotational loading [56]. Hindfoot planovalgus correction induces changes in radiographic parameters of HV, sometimes with paradoxical effects on forefoot deformity progression [74]. Despite overall improvement in alignment, progression of the HVA has been reported in a significant proportion of feet following correction [74]. Moreover, the relationships between hindfoot parameters and HV severity may be weak or non-linear, with some variables not consistently correlating with deformity magnitude [55]. Overall, these findings suggest that biomechanical interactions across the hindfoot, midfoot, and forefoot are complex and non-linear, and that isolated correction of either the forefoot or hindfoot may not capture the full extent of first ray rotational changes. They highlight the need for a global, medial column–based approach to three-dimensional foot deformities [53–57, 74]. Accordingly, various surgical strategies have been developed to correct the deformity across all three spatial planes. Distal and proximal osteotomies allow multiplanar correction of the first ray, whereas Lapidus-type arthrodesis is particularly indicated in cases of TMT instability, providing global correction that includes axial, sagittal, and rotational components [87–91].

The integration of the rotational component has modified the assessment of surgical outcomes. The inability of Scarf osteotomy to correct first metatarsal pronation has been demonstrated on WBCT, with no significant change in the MPA (11.4 ± 7.7° preoperatively vs 11.4 ± 9.9° postoperatively; p = 0.75) or the alpha angle (10.9 ± 8.0° vs 10.7 ± 13.1°; p = 0.83) [92]. In contrast, conventional radiographic parameters showed significant improvement, including reductions in the HVA (28.6 ± 10.1° to 12.1 ± 7.7°; *p* < 0.001) and IMA (13.7 ± 3.8° to 7.5 ± 3.0°; *p* < 0.001), highlighting persistent coronal plane malrotation despite transverse plane correction. Moreover, higher postoperative MPA and alpha angle values were significantly correlated with worse functional outcomes (MOXFQ: Manchester-Oxford Foot Questionnaire and VAS: Visual Analog Scale for pain), with correlation coefficients of r = 0.76 and r = 0.67 [92]. These findings confirm that Scarf osteotomy does not reliably correct first metatarsal rotation and that residual pronation is associated with inferior clinical outcomes, supporting the need to address the rotational component in surgical planning. These data further suggest that the proposed pathological thresholds for three-dimensional parameters, particularly the α angle and the MPA (Table 5), may play an important role in surgical decision-making. Values exceeding these thresholds support consideration of a planned three-dimensional rotational correction or a Lapidus procedure rather than a strategy based solely on conventional two-dimensional radiographic planning.

These findings support techniques providing more effective rotational correction, particularly proximal osteotomies or the modified Lapidus procedure. In a WBCT-based series, the Lapidus procedure significantly reduced first metatarsal pronation from 29.0 ± 8.7° preoperatively to 20.2 ± 5.4° postoperatively (− 8.8°; *p* < 0.001) [90], independent of sesamoid position. The IMA decreased from 16.7 ± 3.2° to 8.8 ± 2.8° (*p* < 0.001), confirming transverse and rotational correction [90]. However, a systematic review (> 16,000 procedures) reported recurrence (~ 4.9%), persistent MTP pain (1.5%), dissatisfaction (10.6%), transfer metatarsalgia (6.3%), infections (2.6%), and reoperations (~ 2.1%), with variable nonunion rates, generally higher than other procedures [93]. Combined techniques are therefore proposed to reduce complications while preserving biomechanical benefits of TMT stabilization. Lapidus arthrodesis may induce morphometric changes. In a 32-foot series, relative metatarsal shortening averaged − 4.1 mm (*p* < 0.0001), with only one case of transfer metatarsalgia, suggesting limited clinical impact. Radiographic improvements included reductions in IMA (15° to 5°) and HVA (33° to 9°; *p* < 0.0001) [94]. The LapiCotton technique, combining TMT arthrodesis and Cotton osteotomy, restores medial column stability. In 22 patients, it improved sagittal alignment (talus–first metatarsal angle + 9.4°; *p* < 0.0001), with 91% fusion at 3 months and low complication rates (9% minor, 4.5% major), supporting early radiographic correction pending long-term validation [95].

## Current and future perspectives

WBCT has significantly improved the three-dimensional characterization of HV by enabling a detailed and comprehensive analysis of the different components of the deformity. However, the richness of the generated data also represents a limitation, due to the time required for analysis and the level of expertise needed for interpretation. The most accurate three-dimensional measurements still frequently rely on manual or semi-automated segmentation of osseous structures, requiring dedicated surface or volumetric landmarks [96]. In this context, computer-assisted analysis tools have been evaluated in patients with HV, showing that semi-automated approaches provide accuracy comparable to manual measurements while maintaining good discriminatory performance between pathological and healthy feet. In a WBCT study, both methods showed excellent reproducibility (ICC > 0.80), with strong agreement between semi-automated and manual measurements (r = 0.81 for HVA, 0.90 for IMA, 0.66 for IPA; *p* < 0.001) and mean differences below 3.2° [97]. These findings support the integration of semi-automated methods into clinical WBCT analysis.

The rise of artificial intelligence (AI) and automated segmentation techniques represents a major development in the analysis of foot deformities [98]. Deep learning algorithms have already demonstrated the ability to automatically extract HV parameters from conventional radiographs with performance comparable to expert assessment. In a recent study, automated measurements achieved excellent agreement with clinician measurements for the HVA (ICC = 0.97) and good-to-excellent agreement for the IMA (ICC = 0.75–0.89), while automated treatment recommendations showed approximately 80% concordance with expert surgical decision-making [99]. However, their application to WBCT data remains to be developed, due to the three-dimensional complexity of osseous structures and joint interactions. In recent three-dimensional WBCT-based approaches, quantitative methods such as distance mapping and coverage mapping enable detailed assessment of first ray articular relationships. Distance mapping quantifies three-dimensional inter-surface distances between the first metatarsal, proximal phalanx, and sesamoids, while coverage mapping converts these data into color-coded topographic representations of articular contact and uncoverage [100]. In a case–control study including 44 patients with HV and 43 controls, coverage mapping revealed a significant reduction in first metatarsophalangeal joint coverage (42.7% vs 50.1%, *p* < 0.001) and marked decreases in medial and lateral metatarsosesamoid coverage (31.1% vs 54.4% and 22.6% vs 46.0%, respectively; *p* < 0.001) [100]. Quadrant analysis showed regional redistribution of contact consistent with joint subluxation. Interobserver reproducibility was excellent (ICC 0.832–0.854) [100], whereas distance mapping alone showed no significant group differences, underscoring the higher sensitivity of coverage mapping.

Beyond morphologic assessment, WBCT-derived datasets may also support biomechanical modeling approaches such as finite element analysis (FEA), allowing evaluation of forefoot loading conditions and stress distribution under physiological and pathological states [101, 102]. These models have demonstrated altered biomechanics in HV, including changes in metatarsal stress distribution and joint loading patterns, and may further enable virtual simulation of corrective surgical procedures and their impact on forefoot mechanics [101]. In addition, the development of patient-specific 3D-printed cutting guides represents a logical extension of WBCT-based surgical planning. By integrating three-dimensional reconstructions derived from WBCT data, these tools may improve the accuracy of multiplanar first ray correction, particularly for rotational deformities that are difficult to control with conventional techniques [103]. Early evidence suggests that 3D-printed models can influence surgical planning and correction strategies, especially regarding first metatarsal rotation, supporting their potential role in HV surgery [103]. Overall, these developments suggest a transition toward more automated, standardized, and reproducible WBCT-based analysis tools. In the long term, these advances may improve the morpho-functional understanding of HV, optimize therapeutic planning, and contribute to better prediction of clinical outcomes.

## Conclusion

WBCT has significantly advanced the understanding of HV by enabling a comprehensive three-dimensional assessment of the first ray under physiological loading conditions. The literature consistently demonstrates that HV is not a simple coronal plane deformity, but rather a triplanar disorder involving metatarsal pronation, TMT instability, sesamoid displacement, and complex interactions within the medial column and hindfoot.

These insights highlight the limitations of conventional radiography in capturing the rotational and multiplanar components of the deformity and underscore the added value of WBCT in both morphological assessment and surgical planning. Furthermore, emerging developments in semi-automated segmentation, artificial intelligence (AI), and advanced three-dimensional mapping techniques are expected to improve reproducibility, standardization, and clinical translation of WBCT analysis.

Together, these advances position WBCT as a key imaging modality for a more precise, functional, and individualized evaluation of HV.

## Data Availability

Not applicable.

## Abbreviations

CT: Computed Tomography
DMAA: Distal metatarsal articular angle
HVA: Hallux valgus angle
HV: Hallux valgus
IMA: Intermetatarsal angle
MPA: Metatarsal pronation angle
TMT: First tarsometatarsal joint
WBCT: Weightbearing computed tomography
